# Targeting Metabolism to Control Immune Responses in Cancer and Improve Checkpoint Blockade Immunotherapy

**DOI:** 10.3390/cancers13235912

**Published:** 2021-11-24

**Authors:** Angèle Luby, Marie-Clotilde Alves-Guerra

**Affiliations:** Institut Cochin, Université de Paris, INSERM, CNRS, F-75014 Paris, France; angele.luby@inserm.fr

**Keywords:** immune response, cancer, metabolism, immunotherapy, metabolic drug

## Abstract

**Simple Summary:**

In a tumor context, antitumor immune cells mediate an inflammatory response after activating a metabolic switch to kill cancer cells. However, tumors develop strategies to avoid destruction. Cancer cells are able to modify the metabolic environment of the tumor by sequestering nutrients (e.g., glucose, tryptophan, arginine) and by producing toxic waste compounds (e.g., adenosine, lactate, kynurenine). This tumor environment promotes exhaustion of antitumor immune cells while driving the expansion of Tregs and the expression of immune checkpoints. Establishment of such an immunosuppressive tumor environment decreases treatment response of cancer patients to immunotherapy. Interestingly, immunometabolism knowledge allows new therapeutic strategies to increase antitumor immune response by targeting the metabolism of both cancer and immune cells to improve immunotherapy.

**Abstract:**

Over the past decade, advances in cancer immunotherapy through PD1–PDL1 and CTLA4 immune checkpoint blockade have revolutionized the management of cancer treatment. However, these treatments are inefficient for many cancers, and unfortunately, few patients respond to these treatments. Indeed, altered metabolic pathways in the tumor play a pivotal role in tumor growth and immune response. Thus, the immunosuppressive tumor microenvironment (TME) reprograms the behavior of immune cells by altering their cellular machinery and nutrient availability to limit antitumor functions. Today, thanks to a better understanding of cancer metabolism, immunometabolism and immune checkpoint evasion, the development of new therapeutic approaches targeting the energy metabolism of cancer or immune cells greatly improve the efficacy of immunotherapy in different cancer models. Herein, we highlight the changes in metabolic pathways that regulate the differentiation of pro- and antitumor immune cells and how TME-induced metabolic stress impedes their antitumor activity. Finally, we propose some drug strategies to target these pathways in the context of cancer immunotherapy.

## 1. Introduction

Thanks to a renewed interest in the last decade, tumor metabolism is now well characterized [1]. Since the 1920s, when Otto Warburg highlighted an increase in aerobic glycolytic activity in cancer cells, different discoveries have shown that tumor metabolism is a complex network of rewiring biochemical reactions allowing the anabolic growth of cancer cells by the conversion of nutrients into metabolites [2,3,4]. Nutrients such as glucose, amino acids or fatty acids are metabolized through glycolysis and/or the tricarboxylic acid (TCA) cycle to be converted into adenosine triphosphate (ATP), proteins, lipids and lactate to support the energy and “building block” demands of highly proliferative cells [4,5,6]. Aberrant mutations enable tumor cells to acquire this metabolic state, and the most studied aberrant mutations belong to the myelocytomatosis oncogene (MYC) and phosphoinositide 3-kinase (PI3K)–protein kinase B (Akt)–mammalian target of rapamycin (mTOR) signaling pathways. Under hypoxic conditions, stabilization of hypoxia-inducible factor-1-alpha (HIF-1α) enhances overexpression of the PI3K–Akt–mTOR pathway and glucose transporters (e.g., GLUT-1) to reinforce glucose consumption and acidification of the tumor microenvironment (TME) [6,7,8,9].

**Figure 1:**
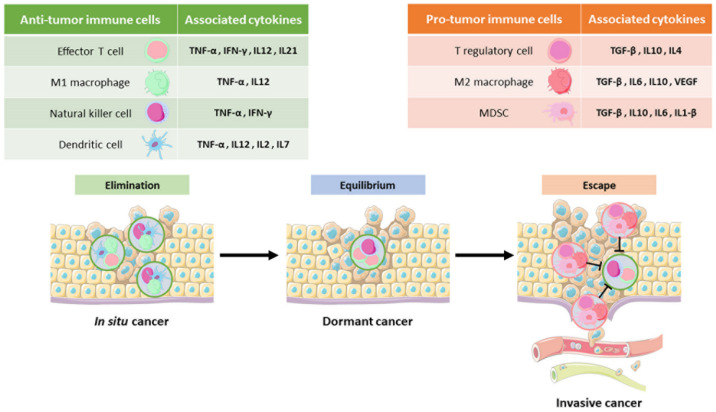
The 3 phases of the immunoediting process in cancer. Once solid tumors reach a defined size, inflammatory signals attract innate immune cells (dendritic cells, M1 macrophages and natural killer cells) which then activate effector T cells by the secretion of associated cytokines (e.g., TNF-α, IFN-γ, IL2, IL7). This process called immunosurveillance (left) detects and eliminates tumor cells by releasing cytotoxic molecules. The tumor then enters an equilibrium phase (middle) in which its growth is maintained by the secretion of IFN-γ but not sufficiently to avoid the appearance of mutations. The cells then become uncontrolled; this is the setting up of the escape process (right). The recruitment of immunosuppressive cells (MDSC, M2 macrophages and T regulatory lymphocytes), through the release of protumor mediators (e.g., TGF-β, IL4, IL10, VEGF), contributes to the uncontrolled growth of the cancer and to the exhaustion of antitumor cells. IFN-γ: interferon γ; IL: interleukin; M1: macrophage 1; M2: macrophage 2; MDSC: myeloid-derived suppressor cell; TGF-β: tumor growth factor β; TNF-α: tumor necrosis factor α.

The “hallmarks of cancer” were updated in 2011 by Hanahan and Weinberg to include cancer metabolic reprogramming and immune escape in the definition of cancer progression. This has created a new impetus for research into immunotherapy and the metabolic crosstalk between immune and tumor cells [1]. Indeed, it has been shown through a significant number of studies that both activated immune cells and cancer cells rely on the same metabolic pathways leading to resource competition for key nutrients [10,11,12]. As a consequence, immune cells, in unfavorable conditions to fight the powerful machinery of cancer cells, will consume toxic nutrients modifying many intracellular metabolic pathways that alter their effector functions and promote tumor invasion. Metabolic modifications directly participate in the establishment of the three successive steps of the immunoediting process taking place in cancer (Figure 1). The first step is the setting of immunosurveillance mechanisms that detect and eliminate tumor cells through the antigen-presenting cells (APCs). Tumor-infiltrating lymphocytes (TILs), proinflammatory macrophages (M1) or natural killer (NK) cells, relying on glycolytic metabolism, infiltrate the tumor and attempt to eradicate it with antitumor responses. The second step takes place when cancer cells survive with the establishment of an equilibrium phase favoring immune metabolic exhaustion. These alterations contribute to the third step characterized by immune escape with immunosuppressive actors. Protumor lymphocytes (Tregs), anti-inflammatory macrophages (M2) and myeloid-derived suppressor cells (MDSCs) which survive in this restrictive environment thanks to their oxidative metabolism are then recruited to encourage tumor growth and exhaustion of antitumor immune cells. In addition, tumor cells also set up immune checkpoints (e.g., PDL1) to directly contest antitumor immunity [13,14,15]. Moreover, immune escape is promoted by hypoxia, glucose depletion and toxic waste product (e.g., lactate and kynurenine) enrichment in the TME [10,11,16]. All these environmental conditions cause inhibition of tumor antigen presentation by APCs and a decrease in the fitness of all antitumor immune cells (e.g., T effector, NK, M1), whereas protumor immune cells (Treg, M2 and MDSC) proliferate and expression of inhibitory checkpoint ligands (e.g., PD1) increases on the surface of immune cells to inhibit antitumor immunity [11,17].

Therefore, one of the current major challenges in cancer therapy is to find an effective tool or combination of tools to reactivate the immune defense to eradicate tumor cells. Many studies have already been done targeting metabolic changes to impact cancer progression without satisfactory results. In addition, there are currently immunotherapy strategies based on the use of monoclonal antibodies that neutralize immune checkpoints, but again the success of these treatments is limited to some patients and types of cancer [18]. To improve cancer treatment efficiency, novel promising approaches include the combination of metabolic targeting of immune cells and cancer cells with immune checkpoint blockade (ICB) [19].

In this review, we focus our interest on the two most common immune cell types studied and explored in cancer immunity research: T cells (T effector and Treg) and macrophages (M1 and M2). First, we summarize new discoveries on activation, polarization and metabolic differentiation of these immune cells in the TME. Then, we highlight the impact of nutrient deprivation and deleterious metabolite production on tumor-associated immune cells, with a special focus on metabolic fitness, exhaustion and antitumoral function. Lastly, we provide some innovative therapeutic strategies using key metabolic targets to reprogram immune cell metabolism to improve immunotherapies.

## 2. Pro- and Antitumor Immune Responses

Antitumor immune cells have a central role in tumor eradication. These immune cells mediate antitumor functions through the lysis of tumor cells and the secretion of inflammatory factors. Despite the powerful antitumor activity of immune cells, tumors develop strategies to avoid their destruction, in particular through the establishment of a metabolic environment that favors the activity of protumor immune cells. In the past 10 years, studies have emerged showing the importance of the metabolism of immune cells (e.g., T cells, macrophages, dendritic cells), which has been shown to be closely associated with their status and functions. Understanding the relationship between the metabolic status and functional differentiation of immune cells is thus necessary to develop new immunomodulatory therapies against cancer.

### 2.1. Metabolic Reprograming of T Cells

T lymphocytes are a heterogeneous group of cells (CD8^+^ and CD4^+^) derived from thymopoiesis producing naive T cells. The maturation of thymocytes into naive T cells occurs through successive steps of expression and repression of cell surface markers. Immature thymic populations, also called double-negative cells (DNs), do not express either the CD4 or CD8 marker. Instead, they are characterized by a CD44^+^CD25^+^ phenotype. Interleukin 7 (IL7), secreted by stromal cells in the thymus, maintains the survival of DNs by upregulating the antiapoptotic protein B-cell lymphoma-2 (BCL-2) and supports metabolic needs of DNs by increasing the expression of amino acid transporter (CD98) and transferrin (CD71). Then, only the cells that successfully undergo a first rearrangement of the TCR (β-selection) and the subsequent loss of the CD44 and CD25 markers are preserved. The expression of a pre-T cell receptor (TCR) allows the formation of a complex with the CD3 molecule which positively regulates the expression of CD4 and CD8. At this intermediate stage, thymocytes are double-positive (DP) (CD4^+^CD8^+^) and are engaged in active proliferation which requires a metabolic change. Notch 1 increases the glycolytic flux in DP through phosphorylation of PI3K and transcription of its target genes. Following a second arrangement of the TCR (α-selection), cells are positively selected as naive CD4^+^CD8^−^ or CD4^−^CD8^+^ T cells through an interaction with an autoantigen–major histocompatibility complex (MHC) which determines the lymphocyte phenotype [20,21,22,23]. Naive T cells display a quiescent metabolism associated with a slow rate of proliferation to survive with low energy demands. They produce ATP through oxidative phosphorylation (OXPHOS) and fatty acid oxidation (FAO) controlled by the transcription factor forkhead box protein O1 (FOXO1) and IL7 activation [24,25]. In addition, these cells maintain low glycolytic activity by repressing mTOR signalization via tuberous sclerosis (Tsc1) expression [26,27]. After being subjected to DN and DP selection in the thymus, T cells migrate to the periphery and enter the naive T cell compartment. In the periphery, naive T cells continuously recirculate between secondary lymphoid organs (spleen and lymph nodes) and blood until they recognize specific antigens with their TCR.

The presentation of antigens to T cells, by MHC (I or II) of antigen-presenting cells (APCs), engages TCR signaling associated with costimulatory signal. The costimulatory signal involves the interaction of the CD80 protein expressed by APCs with the CD28 receptor expressed by lymphocytes (CD4^+^ and CD8^+^ naive T cells). It allows the formation of the immunological synapse which can then induce the secretion of cytokines. The profile of secreted cytokines will directly define the differentiation of T cells (effector T cells (CD4^+^ helper and CD8^+^) and T regulatory lymphocytes (Tregs). CD8^+^ T cells, also known as cytotoxic T lymphocytes (or CTLs) when they are active, have the central function of killing cancer cells by releasing granzyme and perforin. CD4^+^ helper T cells have a “war leader” role. They release anti-inflammatory cytokines to recruit and activate other immune cells. Finally, T regulatory cells are a subtype of immunosuppressive CD4^+^ T cells. Their mission is to maintain self-tolerance and thus promote cancer progression by inhibiting antitumor immunity.

**Figure 2:**
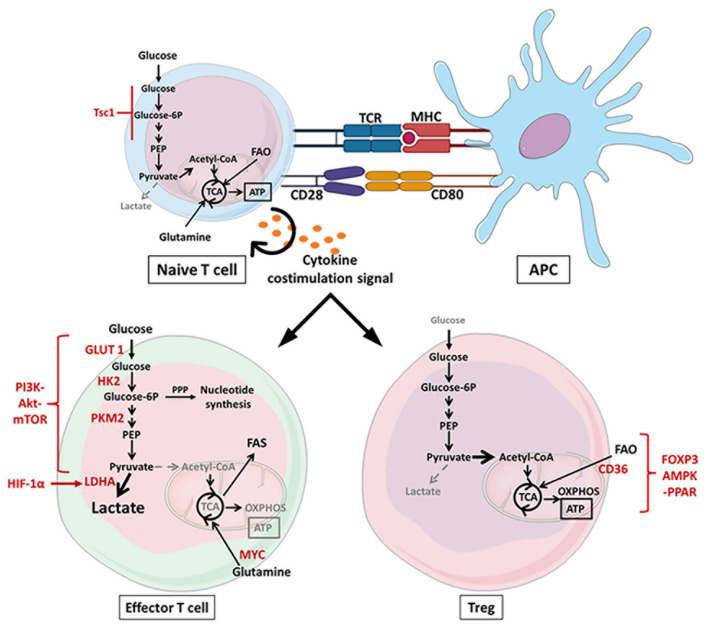
Activation of lymphoid cells through metabolic modifications. Naive T cells have a quiescent metabolism that depends on oxidative phosphorylation. Repression of the mTOR pathway by the Tsc1 gene inhibits glycolytic activity but supports ATP synthesis through oxidation of fatty acids (FAs) and glutamine in the tricarboxylic acid (TCA) cycle. Presentation of antigens to T cells, by the MHC of the antigen-presenting cell (APC), engages T cell receptor (TCR) signaling associated with CD28–CD80 costimulatory receptors and cytokine secretion. The succession of these 3 activation signals determines the differentiation and metabolic profiles of T lymphocytes. Proinflammatory cytokines (e.g., IFN-γ, IL6, IL12) induce glycolytic genes (GLUT1, HK2, PKM2, LDHA) through upregulation of the PI3K–Akt–mTOR, MYC and hypoxia-inducible factor 1α (HIF-1α) signaling pathways. The glycolytic switch produces ATP by converting pyruvate to lactate and supports the biosynthesis of nucleotides through the pentose phosphate pathway (PPP) and lipids through the fatty acid synthesis (FAS). At the same time, anti-inflammatory cytokines (e.g., IL4, TGF-β) promote the establishment of an oxidative metabolism. Differentiated Tregs through FOXP3–AMPK–PPAR signaling upregulate the lipid transporter CD36 to oxidize lipids and thus support mitochondrial functions. Akt: protein kinase B; AMPK: AMP-activated protein kinase; ATP: adenosine triphosphate; APC: antigen-presenting cell; FAO: fatty acid oxidation; FAS: fatty acid synthase; FOXP3: forkhead box P3; GLUT1: glucose transporter 1; HIF-1α: hypoxia-inducible factor 1α; HK2: hexokinase 2; IFN-γ: interferon γ; IL: interleukin; LDHA: lactate dehydrogenase A; MHC: major histocompatibility complex; mTOR: mammalian target of rapamycin; NO: nitric oxide; PI3K: phosphoinositide 3-kinase; PKM2: pyruvate kinase M2; PPAR: peroxisome proliferator-activated receptor; PPP: pentose phosphate pathway; TCA: tricarboxylic acid; TCR: T cell receptor; TGF-β: tumor growth factor β; Treg: T regulatory lymphocyte.

Antitumor cytokines such as tumor necrosis factor α (TNF-α), interferon γ (IFN-γ) and IL12 lead to metabolic reprogramming of effector T cells through activation of glycolytic signaling pathways such as PI3K–Akt–mTOR [28,29,30]. This glycolytic switch allows fast ATP supply, regeneration of nicotinamide adenine dinucleotide (NAD^+^) and nucleotides synthesis required for effector activity, cytokine production and cell proliferation. Autocrine secretion of IL2 increases glucose transporter 1 (GLUT1) expression and enhances PI3K pathway activity [28,31]. Engagement of the TCR leads to the downregulation of negative regulators of PI3K signaling (PTEN and PIK3IP1/TrIP), facilitating metabolic reprogramming [32,33]. PI3K phosphorylates Akt which maintains high expression of GLUT1 to transport glucose and controls glycolytic enzymes such as hexokinase (HK2), pyruvate kinase (PKM2) and lactate dehydrogenase (LDHA) [28,34]. Moreover, the activation of mTOR and cMyc increases HIF-1α levels to reinforce glycolysis and decreases oxygen consumption rate (OCR) of CD8^+^ T cells [35]. Therefore, deletion of HIF in CD8^+^ T cells reduces their infiltration in tumors and accordingly increases tumor growth [36]. A recent study further detailed the glycolysis process associated with the activation of CD4^+^ and CD8^+^ T cells. They highlighted two different phases: first, minutes after TCR engagement, rapid glycolysis takes place and is mediated by pyruvate dehydrogenase kinase 1 (PDHK1) which inhibits OXPHOS but promotes lactate production independently of glucose uptake. Then, in a second step, CD28, PI3K pathway and HIF-1α activation will maintain a high rate of glycolysis [37] (Figure 2).

Although the glycolytic flux is predominant, OXPHOS still remains functional. Amino acids can be used as a source of fuel for the TCA cycle and as precursors for protein synthesis. Therefore, T cell activation increases glutamine uptake thanks to the amino acid transporter 2 (ASCT2). Glutamine, which is the main amino acid in blood, can enhance mTOR expression and provides α-ketoglutarate to the TCA through glutaminolysis to support Th1 and Th17 proliferation and IL2 and IFN-γ secretion [38,39,40,41]. Serine has also been shown to be essential for CD8^+^ T cell proliferation through de novo purine biosynthesis [42]. Moreover, T cells increase fatty acid synthesis to sustain new cell membrane building. In fact, several studies reported that acetyl-CoA carboxylase 1 (ACC1)-mediated de novo lipogenesis is a key process to induce Th17 and CD8^+^ T cell expansion [43,44,45].

However, when the tumor environment becomes poor in nutrients, especially in glucose, Tregs gain the upper hand over other T cells. Under tumor growth factor β (TGF-β) secretion, forkhead box P3 (FOXP3)—a differentiation marker of Tregs—leads to downregulation of glycolysis resulting in increased OXPHOS [46]. Angelin et al. demonstrated that FOXP3 downregulated MYC expression by directly binding the Myc promoter in Tregs [47]. The inhibition of MYC-related glycolysis was supported by the stabilization of the transcription factor FOXO1 which promoted the decrease in Akt and the activation of PTEN. These results have been confirmed with the use of rapamycin, an mTOR inhibitor, which increased the Treg proliferation rate [48]. Nevertheless, a recent study has shown that Tregs could utilize the glycolytic flux to the same extent as Th17 T cells, although they are not dependent on glycolysis to proliferate [43]. However, in a low-glucose and high-lactate tumor environment, Tregs are able to oxidize lactate to pyruvate and to upregulate CD36 expression to oxidize lipids. The interaction between peroxisome proliferator-activated receptor-β (PPAR-β) and fatty acid receptor CD36 (a central metabolic regulator) stimulates FAO to sustain mitochondrial fitness and electron transport chain (ETC) function. Accordingly, deletion of CD36 induces high apoptosis combined with a metabolic shift in Tregs, and therefore this leads to a decrease in tumor growth in Treg^CD36−/−^ mice engrafted with melanoma [49]. Under metabolic stress conditions, activation of AMP-activated protein kinase (AMPK) strengthens FAO by inhibiting key enzymes of lipid synthesis (ACC1 and ACC2) and also suppresses glycolytic transcription factors mTOR and HIF-1α [44,50,51]. Furthermore, under conditions of Th17 polarization and in the presence of an ACC inhibitor, naive T cells differentiate into a Treg phenotype by increasing FOXP3 expression and AMPK levels [44] (Figure 2).

Following an infectious disease or cancer, antigens are cleaned up and only a few memory T cells survive. These memory T cells also present a metabolic switch that depends on the structure of mitochondria. Indeed, memory T cells have elongated, fused and large mitochondria compared to the mitochondria of effector T cells. The protein Opa1 is required to keep cristae organized, fuse the mitochondrial network and stabilize mitochondrial DNA. The phenotype engaged by Opa1 expression promotes OXPHOS and FAO [52,53]. In comparison, the mitochondrial profission protein dynamin-1-like (Drp1) is required for the metabolic reprogramming of effector T cells and their expansion upon activation [54]. Finally, although memory T cells and naive T cells have similar metabolisms, naive T cells have a lower mitochondrial mass and consequently a less active metabolism allowing them to discriminate their respective functions [53].

### 2.2. Metabolic Reprogramming of Macrophages

**Figure 3:**
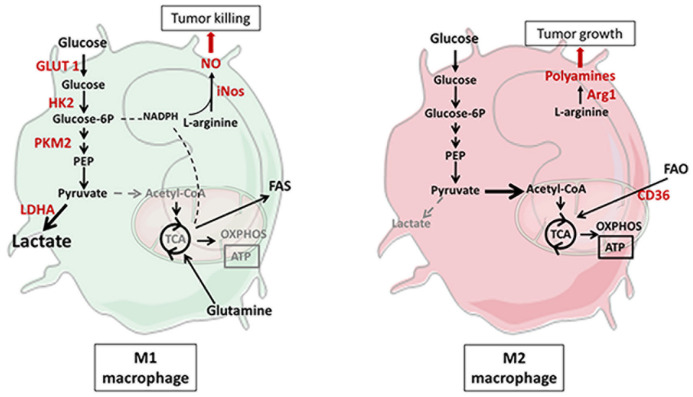
Activation of myeloid cells through metabolic modifications. In polarized M1 macrophages, the PI3K pathway and overexpression of HIF-1α promote glycolysis via an increase in glycolytic enzymes GLUT1, HK2, PKM2 and LDHA. Glutamine is metabolized in the TCA cycle to support the synthesis of FAs and nicotinamide adenine dinucleotide phosphate (NADPH). NADPH is then converted by inducible nitric oxide synthase (iNOS) into L-arginine and nitric oxide (NO) which play a major role as cytotoxic effector molecules against tumor cells (**left**). Like Tregs, the proliferation of polarized M2 macrophages is supported by FA oxidation. Lipids carried by the CD36 transporter are oxidized through the TCA cycle to produce ATP through oxidative phosphorylation (OXPHOS). In M2, the enzyme arginase 1 (Arg1) converts L-arginine to polyamine to support tumor growth (**right**). ATP: adenosine triphosphate; APC: antigen-presenting cell; Arg1: arginase 1; FAO: fatty acid oxidation; FAS: fatty acid synthase; GLUT1: glucose transporter 1; HIF-1α: hypoxia-inducible factor 1α; HK2: hexokinase 2; iNOS: inducible nitric oxide synthase; LDHA: lactate dehydrogenase A; M1: macrophage 1; M2: macrophage 2; MHC: major histocompatibility complex; NADPH: nicotinamide adenine dinucleotide phosphate; NO: nitric oxide; PKM2: pyruvate kinase M2; TCA: tricarboxylic acid.

Myeloid cells are also involved in cancer immunity. Macrophages, derived from circulating monocytes, participate in the phagocytosis of dying cells and the secretion of cytokines. They can also present tumor peptide antigens to naive T cells through MHC. Like T lymphocytes, activated macrophages are polarized into different subtypes. The metabolic switch undergone during infections or cancers determines if macrophages will be differentiated into either a classical antitumor M1-like phenotype or an alternative immunosuppressive M2-like phenotype. The metabolic pattern will define the polarization of macrophages and influence the prognosis of cancer patients [55,56]. M1-like cell activation is induced by proinflammatory stimuli such as lipopolysaccharides (LPSs) and INF-γ which boost glycolysis [57]. Usually, the glycolytic metabolism of tumor-infiltrating M1 macrophages is associated with a good prognosis and regression of the tumor mass; however, different publications have shown that M1 macrophages also promote malignant transformation and metastasis [58,59]. Like T cells, activation of PI3K signaling pathway and overexpression of HIF-1α in M1 macrophages promote glycolysis by upregulation of glycolytic enzymes such as GLUT1, PKM2 and HK2 [57,60,61,62] (Figure 3). Recently, a study demonstrated that 2-deoxy-D-glucose (2-DG), a glycolysis inhibitor, prevented M1 macrophage differentiation by decreasing GLUT1 expression, proton production rate and cytokine secretion [63]. The glycolytic switch and the inhibition of the TCA cycle associated with the inhibited phosphorylation of pyruvate dehydrogenase (PDH) lead to glucose metabolic rewiring towards the pentose phosphate pathway (PPP) [64,65]. Some studies have shown that PPP is useful in inflammatory macrophages to increase levels of nicotinamide adenine dinucleotide phosphate (NADPH) which catalyzes reactive oxygen species (ROS) production by NADPH oxidase [66]. Moreover, proinflammatory factors (e.g., LPS, IFN-γ, TNF-α) stimulate the catabolism of arginine to produce nitric oxide (NO) via inducible nitric oxide synthase (iNOS). In 2019, Bailey et al. showed that macrophages activated in vitro by IFN-γ have an inhibition of the activity of ETC depending on iNOS, while the increase in glycolytic metabolism and lactate production is unchanged in KO iNOS macrophages [67]. Both ROS and NO play a major role as cytotoxic effector molecules against tumor cells. High expression of iNOS has been reported to be linked to good prognosis in ovarian and lung cancers and was also associated with low metastasis numbers in a pancreatic cancer xenograft model in mice [68,69,70].

In tumors, tumor-associated macrophages (TAMs) have been shown to express an M2-like phenotype. Indeed, the oxidative metabolism of protumoral M2-like macrophages promotes their expansion to the detriment of M1. In response to anti-inflammatory cytokines such as IL4 and IL10 and glucocorticoid stimulations, M2 cells acquire their immunosuppressive phenotype by undergoing a metabolic switch. They increase their OCR to support their proliferation [71] (Figure 3). Their oxidative metabolism is sustained by an increase in the number of mitochondria and their mitochondrial content, reflected by an upregulation of succinate dehydrogenase A (SDHA) [63,71,72]. Thus, treatment of M2 macrophages with an SDHA inhibitor affected M2 homeostasis by inducing the downregulation of protumor factors, OCR levels and mitochondrial mass [63]. In the M2 polarization context, FAO is the main source of fuel that supplies the TCA cycle. IL4 stimulation induces crosstalk between signal transducer and activator of transcription (STAT6) and PPAR-γ, which in turn upregulates CD36 expression to bind and translocate fatty acids in cells [71,73,74]. Several studies proved that lipid oxidation is essential for M2-like TAM differentiation. Indeed, FAO is required for migration, proliferation and tumor invasion via IL-1β secretion. Moreover, macrophages without CD36 or PPAR-γ acquired an antitumor phenotype [71,75,76]. Additionally, PPAR-γ^−/−^ M2 macrophages had lower mitochondrial content and decreased expression of the M2 differentiation marker arginase 1 (Arg1) [77]. Unlike M1 macrophages that metabolize L-arginine by NOS2 to produce NO and kill tumor cells, M2-TAMs convert arginine to polyamines by activating expression of ARG1. This activity of ARG1 promotes tumor growth, metastasis and neovascularization, and it is associated with poor prognosis in different cancer types [78,79,80,81].

While it is now clear that M2 macrophages have enhanced FAO and OXPHOS metabolism, the involvement of glycolysis in their metabolic switch is still debated [82,83]. The most recent literature does not show an increase in GLUT1 expression and in the rate of proton production when macrophages are polarized with IL4 [63]. Moreover, glucose deprivation or glucose substitution with galactose did not affect M2 differentiation as OXPHOS remains active. Wang et al. have indeed demonstrated that STAT6–PPAR-γ–CD36 signaling was not altered in macrophages derived from bone marrow stimulated with IL4 in galactose medium [84]. However, some older studies demonstrated that glucose oxidation was required for M2 differentiation. They showed that glycolysis was enhanced in M2 through Akt–mTOR signaling pathway inducing the expression of M2 macrophage specific genes such as Arg1 [83]. It was also highlighted that FAO activation via STAT6 was mechanistically dependent on the mTORC2–IRF4 pathway using glucose as substrate [82].

All these studies reveal the metabolic complexity and adaptive capacities of immune cells (T cell and macrophage subsets). They also highlight the therapeutic interest of targeting metabolic pathways of immune cells to modify their function and thus promote tumor remission.

## 3. Metabolic Variation in the TME and Consequences on Immunity

**Figure 4:**
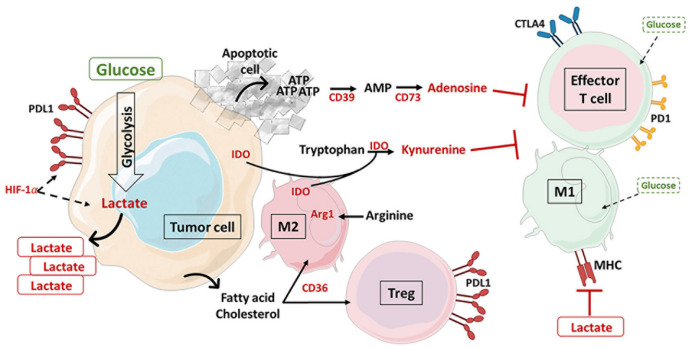
Metabolites produced in the tumor microenvironment (TME) and their impact on antitumor immune cell exhaustion. Metabolic competition with cancer cells affects the supply of nutrients such as glucose and amino acids to antitumor cells, while T regulatory lymphocytes (Tregs) and M2 macrophages (M2) can consume lipids and lactate present in the TME. Many molecules secreted by immune cells or cancer cells participate in immunosuppression. In cancer cells, lactate produced in large quantities by activation of HIF-1α-dependent glycolysis inhibits MHC and consequently the activation of effector T cells. The released ATP is converted by both CD39 and CD37 ectonucleotidases into adenosine which binds to its A2A receptor to affect T cell functions. Indolamine 2,3-dioxygenase (IDO) exerts immunosuppressive functions, both by the deprivation of tryptophan and by the binding of kynurenine on aryl hydrocarbon receptor (AhR). M2 macrophages deplete arginine from the environment via the enzyme arginase 1 (Arg1), affecting cytokine secretion and T cell receptor (TCR) expression. Metabolic changes in the TME promote the expression of immune checkpoints to reinforce the blockade of potential antitumor action, suppress the antitumor response and favor the development of Tregs. AhR: aryl hydrocarbon receptor; AMP: adenosine monophosphate; Arg1: arginase 1; ATP: adenosine triphosphate; CTLA4: cytotoxic T lymphocyte antigen 4; HIF-1α: hypoxia-inducible factor 1α; IDO: indolamine 2,3-dioxygenase; M1: macrophage 1; M2: macrophage 2; MHC: major histocompatibility complex; PD1: programmed cell death protein 1; PDL1: programmed cell death ligand protein 1; TME: tumor microenvironment; Treg: T regulatory lymphocyte.

In order to proliferate, tumor cells must escape the antitumor immune system. Cancer cells are able to alter the tumor metabolic environment by sequestering nutrients and producing compounds that are toxic to the antitumor cells. Thus, chronic TCR stimulation, exposure to metabolic waste products and competition for resources between cancer cells and TILs alter T cell differentiation and lead to T cell exhaustion. The invasion of Tregs into the TME and the expression of immune checkpoints reinforce the development of malignant tumors with poor prognosis [85,86]. This section highlights the involvement of TME metabolism in immunosuppression and tumor progression through different key metabolites (Figure 4).

### 3.1. Glucose–Lactate–Oxygen

The high glycolytic activity of both cancer cells and antitumor immune cells leads to nutrient deprivation in the TME, and limited blood supply creates hypoxic areas especially in the center of the tumor [11]. These particular metabolic conditions can affect immune effector functions including deregulation of TCR signaling. Recent studies have demonstrated a repression of MHC I and II on the surface of cancer cells in oxygen- and glucose-deprived conditions. This downregulation is linked to increased PI3K pathway activity in cancer cell lines and decreased secretion of IFN-γ which is the main regulator of MHC I gene expression [87,88]. Moreover, hypoglycemic TME is able to disturb the mitochondrial metabolism of TILs, leading to their functional exhaustion by accumulation of depolarized mitochondria [85]. However, before total immune escape, CD8^+^ and CD4^+^ T cells are able to transiently adapt their metabolism while maintaining their effector functions. They undergo an oxidative metabolic switch with an increased rate of FAO thanks to the expression of adipose triglyceride lipase (ATGL) and carnitine palmitoyltransferase 1 (CPT1) enzymes. These new metabolic properties, controlled by PD1, assimilate CD8^+^ and CD4^+^ T cells to memory lymphocytes or Tregs. Tumor progression is therefore favored since these cells no longer secrete antitumor cytokines but instead release IL10 which promotes the activation of Treg cells and M2 macrophages [89,90]. Previous studies usually hypothesize metabolic competition between cancer and immune cells, but a pioneering new study shows that glucose is not limited in the TME [91]. On the contrary, the use of positron emission tomography (PET) probes shows that immunosuppressive myeloid cells (MDSC and M2) metabolize more glucose than cancer cells which rather promote the consumption of glutamine. These metabolic adaptations are regulated by intrinsic cellular programs driven by mTOR signaling but not governed by competition for nutrients. Thus, the TME is mainly immunosuppressive and, surprisingly, does not appear to affect the antitumor T cell population. Moreover, rapamycin, a potent inhibitor of the mTOR pathway, affects as expected the glucose consumption of myeloid cells but does not lead to increased proliferation of CD4^+^ and CD8^+^ cells and decreased tumor volume. However, the authors only looked at the glucose consumption of MDSCs and M2 myeloid cells, and it would be interesting to study M1 cells (CD68^+^iNOS^+^) in order to determine whether these intrinsic adaptations are also found in antitumor myeloid populations. Finally, as Reinfeld et al. obtained these results with subcutaneous tumor models, it would be interesting to compare whether the metabolic behavior of immune cells is similar in tumors developed directly in the target organ. Indeed, the tissue vascularization could participate in immune recruitment and nutrient dependency.

Hypoxia alone may also have a direct role in cancer progression, mediated by a key-family factor: HIF. Indeed, HIF-1α will promote M2 differentiation and inhibit M1 infiltration [92]. In MCF-7 breast cancer cells, low oxygen levels decrease the secretion of IFN-γ which allows the activation of effector T cells. On the contrary, they stimulate the PI3K–mTOR pathway to regulate the glycolytic metabolism of cancer cells [90,93]. In addition, HIF-1α increases the expression of PDHK1 which will inhibit PDH by phosphorylation to limit the conversion of pyruvate to acetyl-CoA to fuel the TCA cycle [94]. CD8^+^ T cells initially adapt to hypoxia, and HIF is even necessary for the acquisition of their effector function, but CD8^+^ T cells will be exhausted in the long term and protumor cells will become the majority [95,96].

Moreover, high aerobic glycolysis in tumor cells is automatically coupled with increased release of lactate via monocarboxylate transporters (MCTs), in particular MCT4 in the TME. Lactate-enriched TME altered the activation of macrophages in vitro and in silico in prostate cancer. An acidic tumor was associated with a strong increase in the expression of the Arg1 and CD206 genes related to the tumor-promoting phenotype of TAMs [97]. In melanoma, LDHA expression decreased the expression levels of nuclear factor of activated T cells (NFAT), a transcription factor essential for activation, survival and effector function of T and NK cells via the transcription of IFN-γ. The LDHA^low^ melanoma tumor developed slower than the control tumor and showed increased infiltration of T and NK cells associated with improved mice survival [98]. Interestingly, lactate can impair T cell proliferation independently of microenvironment acidification by inducing reductive stress. Indeed, lactate by elevating the NADH/NAD^+^ ratio during the production of pyruvate depletes the glyceraldehyde-3-phosphate dehydrogenase (GAPDH) and 15-hydroxyprostaglandin dehydrogenase (PGDH) reactions of NAD+ and deprives the proliferating T cells of serine derived from glucose [99].

These TME conditions (glycolytic, hypoxic and acidic) turn out to be a real vicious cycle for patients. Indeed, they favor immunosuppression, a decrease in the response to immunotherapies and the stability of protumor immune cells (Treg and M2) [11,100]. Moreover, tumor-associated macrophages are directly involved in promoting tumorigenesis by increasing aerobic glycolysis of tumor cells, at the expense of T cells. In invasive breast cancer, TAMs secrete extracellular vesicles that stabilize HIF-1α to support high expression of GLUT1 and LDHA glycolysis genes [101]. Zhang et al. highlighted that increased phosphorylation of PGK kinase, which catalyzes 1,3-bisphosphoglycerate to 3-phosphoglycerate, maintained aerobic glycolysis in glioma cells (U87 and U251) cocultured with M2-polarized THP1 cells [102]. Nevertheless, low-glycolysis tumors limit metabolic competition for T effectors, which favors expansion and intratumor infiltration of CD8^+^ T cells. Furthermore, low glycolytic activity potentiates the ability of CTLA4 blockade to induce phenotypic and functional destabilization of Tregs towards IFN-γ-producing cells [100]. As discussed by Zappasodi et al., these results also suggest that the use of anti-CTLA4 could be combined with an inhibitor of tumor glycolysis to be effective on a broad spectrum of tumors.

### 3.2. Lipids

Less studied than glycolytic metabolic reprogramming, lipid metabolism has however emerged in the past few years as a main hallmark of cancer cell deregulation and protumor immune responses. The TME is indeed a lipid-enriched environment. The aberrant synthesis of new fatty acids and cholesterol by cancer cells and the accumulation of adipocytes provide energy substrates, plasma membrane building blocks and signaling molecules to support rapid tumor growth and dissemination of metastases [103,104]. The anabolic rewiring of lipids is regulated by mTORC2 which controls the synthesis of the transcription factor sterol regulatory element-binding protein 1 (SREBP1) which activates the expression of the lipogenesis enzymes FAS and ACC and the formation of lipid droplets [104]. These tumor-associated lipids have been associated with immunosuppressive effects on different types of immune cells. Several studies have revealed an increase in the expression of receptors linked to lipid transport in correlation with the improvement of oxidative metabolism of the protumor infiltrating immune cells. Indeed, the uptake of these lipids is dependent on the translocase of fatty acids CD36 and under the control of the STAT3–STAT5 and PPAR-β signaling in MDSCs and Treg cells, respectively [49,105,106]. Furthermore, CD36–PPAR-β signaling in melanoma has been shown to modulate mitochondrial fitness and levels of the key metabolite NAD to enable the metabolism of lactic acid to pyruvate [49]. Thus, protumor immune cells (Tregs or MDSCs) easily adapt and even strengthen the TME enriched with lactic acid and lipids to maintain their functions, unlike antitumor cells. Lim et al. have successfully shown that the integrity of tumor Tregs is coordinated by sterol regulatory element-binding protein cleavage-activating protein (SCAP)–SREBP signaling [107]. Induction of de novo fatty acid synthesis, cholesterol production (via mevalonate metabolism) and post-transcriptional geranylgeranylation of proteins facilitate the accumulation and immunosuppressive activity of Tregs in tumors through increased expression of PD1 dependent on lipid metabolism adaptations. Thereby, cancer patients exhibited increased amounts of intracellular lipids in dendritic cells which were no longer able to efficiently stimulate T cells because they had acquired defects in tumor antigen processing despite a functional MHC [108]. In CD8^+^ T cells, CD36 is expressed by exhausted cells. This causes excessive accumulation of oxidized intracellular lipids in T cells resulting in lipid peroxidation and ferroptosis, which weakens their antitumor capacity [109,110].

Other recent studies have focused on the direct effects of lipids on T cells. In vivo accumulation of cholesterol and long-chain fatty acids has been observed in the TME of different types of cancers (e.g., breast, melanoma, pancreatic ductal adenocarcinoma). Furthermore, breast cancer progression has been associated with increased levels of 27-hydroxycholesterol, a degradation metabolite of cholesterol produced by the enzyme CYP27A which was able to act as a selective estrogen receptor-α agonist and modify antitumor immune responses [111,112].

Subsequently, two research groups demonstrated an accumulation of intratumoral lipids in CD8^+^ T cells infiltrating pancreatic ductal adenocarcinoma, colon carcinoma and myeloma. These lipid contents (cholesterol and long-chain fatty acids) contributed to metabolic exhaustion of CD8^+^ T cells due to lipotoxicity correlated with increased expression of immune checkpoints (PD1, LAG3, TIM3 and 2B4) and number of Tregs [113,114]. More notably, increased cellular cholesterol specifically upregulated the transcription factor XBP-1 in CD8^+^ T cells. Furthermore, transfer of XBP-1-knockdown CD8^+^ T cells into mice bearing B16 lung tumors induced lower levels of PD1 and 2B4 immune checkpoints compared to control CD8^+^ T cells. XBP-1 activity induced an unfolded protein response and ER stress leading to suppression of mitochondrial activity and increased markers of immune exhaustion [114].

### 3.3. Amino Acids

Depletion of amino acids in the tumor environment is also an adaptive immune phenomenon of cancer fitness.

#### 3.3.1. Tryptophan

Indolamine 2,3-dioxygenase (IDO) is an enzyme involved in the catabolism of tryptophan through the kynurenine pathway. Its production by cancer cells, TAMs, DCs and MDSCs is strongly upregulated in the plasma of patients with many solid and hematological cancers and correlates with a poor patient prognosis [115,116]. Stimulated by IFN-γ, IDO exerts immunosuppressive functions, both by the deprivation of tryptophan and by the production of kynurenine, leading to apoptosis of NK and effector T cells while promoting their differentiation into a regulatory phenotype [117]. The team of I. Stephanis recently provided additional metabolic details on the immunomodulatory mechanism of IDO [118]. On the one hand, the depletion of tryptophan in the TME affects the consumption of glutamine and glucose by immune cells through the activation of the Gcn2 kinase and the inhibition of mTOR. On the other hand, kynurenine, generated by IDO1, binds to the aryl hydrocarbon receptor (AhR) expressed on the surface of T cells. Activated AhR upregulates the expression of PD1 on T cells and supports FAO via PPAR-α and CPT1 activations [118,119]. The interaction between kynurenine and its AhR receptor favors the expansion of Treg cells at the expense of Th17 differentiation [120]. The metabolic changes induced by kynurenine were found to increase β-catenin expression in colon cancer cell lines (HT29 and HCT116) to support cancer proliferation and enhance glucose depletion in the TME [121].

In addition, using pancreatic cancer cells, in vitro and in vivo studies have also shown direct benefits of IDO on cancer cell growth. Indeed, after injection of C13-tryptophan in mice bearing PDAC tumors, liquid chromatography–mass spectrometry (LC-MS) analysis of the tumors highlighted the contribution of tryptophan as a one-carbon source for the tetrahydrofolate cycle for nucleotide synthesis [122].

#### 3.3.2. Arginine

L-Arginine, a semiessential metabolite for mammals, is essential for immune reactions. It is required for central memory-like T cells and participates in the survival, metabolic fitness and antitumor activity of T cells [123]. In a tumor context, MDSCs and M2 macrophages deplete arginine from the environment via the enzyme arginase 1 (Arg1). After activation by IL4 or TNF-α, Arg1 metabolizes arginine to ornithine and urea. These degradation products and arginine depletion affect cytokine secretion and TCR expression [124]. A recent study revealed that STAT3–Arg1 pathway in MDSCs is involved in deregulating the functions of CD8^+^ T cells by decreasing their secretion of granzyme B, perforin and IFN-γ [125]. Moreover, in transgenic mice overexpressing Arg1 with breast cancer, the increased number of Tregs infiltrated into the tumor was correlated with enhanced tumor development [126]. Conversely, arginine supplementation has been shown to improve antitumor immunity and survival in mice with mammary and colon carcinoma xenografts [127,128].

In 2019, an initial study investigating the role of arginine metabolism mediated by the mitochondrial isoform Arg2 in tumor immune responses showed similar results. Indeed, *Arg2*^−/−^ mice controlled tumor growth more efficiently thanks to increased serum arginine levels which induced higher TIL activities [129].

#### 3.3.3. Other Amino Acids

Perhaps less studied, methionine and serine are also required for T cell activation. Proliferating cells depend on mitochondrial one-carbon metabolism to perform biosynthetic functions. The methionine and serine cycles are connected to the tetrahydrofolate cycle to allow the synthesis of amino acids, lipids and glutathione and they promote methylation reactions. Teff expansion leads to a rapid increase in the expression of genes for methionine and serine biosynthesis, especially those which regulate the entry of carbon into the tetrahydrofolate cycle [42]. Cultures combining melanoma and CD8^+^ T cells, by the Transwell system, showed that cancer cells consumed most of the amino acids leading to CD8^+^ mortality. However, methionine supplementation by intratumoral injection in mice bearing B16F10 tumors delayed tumor growth, improved CD8^+^ survival and restored the production of TNF-α and IFN-γ cytokines [130].

### 3.4. Adenosine

In the TME, the levels of extracellular ATP are strongly increased, unlike in physiological situations where they are tightly regulated. In the microenvironment of solid tumors, cells (cancerous, immune, endothelial, stromal, etc.) are subjected to stress, in particular hypoxia and inflammation, which induce an abundant release of intracellular ATP by necrosis and apoptosis. This excess of ATP is first hydrolyzed to AMP and then dephosphorylated to adenosine by the ectonucleotidases CD39 and CD73, respectively. Adenosine exerts its effects by binding to its four known receptor subtypes (A_1_R, A_2A_R, A_2B_R, A_3_R), which are expressed by both cancer cells and immune cells (myeloid and lymphoid lineages) to produce distinct immunosuppressive effects. Adenosine binding activates G proteins which interact with adenylate cyclase to convert ATP to cAMP and thus initiate PKA signaling. Inhibition of NFκB mediated by PKA-dependent phosphorylation of CREB suppresses the secretion of proinflammatory cytokines such as IL2 and IFN-γ, but it promotes secretion of IL10 and TGF-β [131,132]. In addition, Mastelic-Gavillet et al. demonstrated in 2019 that adenosine/A_2A_R signaling affected TCR engagement by deregulating downstream effectors of the mTOR pathway, resulting in impaired glycolytic metabolic capacity in human CD8^+^ T cells [132]. In ovarian cancer, adenosine facilitated the migration and recruitment of infiltrating macrophages and decreased M1 polarization [133]. Furthermore, protumor myeloid cells that express A_2A_R may also participate in immunosuppression by suppressing T and NK cell responses, thereby promoting tumor growth and metastasis [134]. Finally, the coexpression of the two ectonucleotidases (CD39 and CD73) and the adenosine receptor in Tregs contributed to their expansion and to the suppression of antitumor immunity. Indeed, when using an A_2A_R agonist, Tregs upregulated the expression of CTLA4 and PD1 to decrease the effects of immunotherapy [135,136]. In addition, Ohta’s team highlighted the important role of HIF-mediated hypoxia in the regulation of the expression of adenosine-generating enzymes and, consequently, in the remodeling of protumor immunity [137]. The CD39 and CD73 immunosuppressive effects have been extensively studied in solid tumors, but recently similar effects have also been observed in patients with leukemia [138].

It is now evident that modulation of metabolism can alter the immunosuppressive environment of the tumor. Moreover, the establishment of this environment decreases the response of cancer patients to immunotherapy. We therefore propose in the last section some innovative therapeutic strategies using key metabolic targets to reprogram the metabolism of immune cells in order to improve immunotherapies.

## 4. Combination of Metabolic Intervention and Immunotherapy

**Figure 5:**
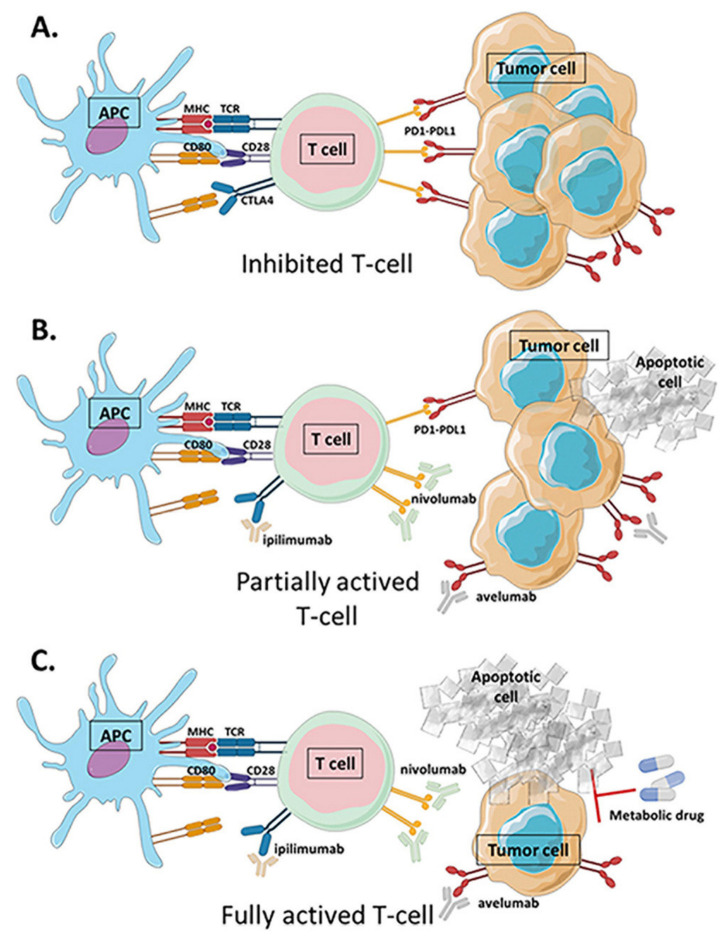
Immune checkpoint blockade strategies to enhance antitumor immune response. (**A**) Immune checkpoints (PD1–PDL1–CTLA4) overexpressed in many tumors inhibit the activating CD28–CD80 costimulation signal of effector T cells and their activity. (**B**) Immune checkpoint blockade (ICB) treatments such as ipilimumab, an anti-CTLA4 antibody; nivolumab, an anti-PD1 antibody; and avelumab, an anti-PDL1 antibody, block the inhibitory binding of immune checkpoints and restore T cell activity but do not lead to cancer remission in many patients. (**C**) Recent strategies propose combining the use of immunotherapy with metabolic drugs to improve the antitumor response. APC: antigen-presenting cell; CTLA4: cytotoxic T lymphocyte antigen 4; MHC: major histocompatibility complex; PD1: programmed cell death protein 1; PDL1: programmed cell death ligand protein 1; TCR: T cell receptor.

**Figure 6:**
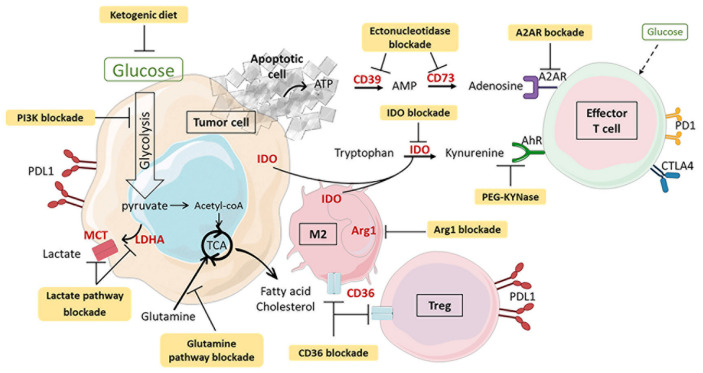
Therapeutic strategies targeting metabolism combined with immunotherapy to enhance the antitumor immune response. The use of small metabolic molecules, antibodies and diet adaptation (in yellow and in bold in the diagram) can modify the metabolic state of the tumor microenvironment (TME). The competition for resources between cancer and immune cells is thus reduced. Tryptophan, arginine, fatty acids and glucose are again available to antitumor immune cells. Targeting of the indolamine 2,3-dioxygenase (IDO), lactate and adenosine pathways prevents deleterious metabolites from suppressing the immune response against cancer. These strategies, which synergize with immunotherapy, must be adapted to the type and stage of cancer and to the interindividual variability of drug response. AhR: aryl hydrocarbon receptor; AMP: adenosine monophosphate; Arg1: arginase 1; ATP: adenosine triphosphate; CTLA4: cytotoxic T lymphocyte antigen 4; IDO: indolamine 2,3-dioxygenase; M1: macrophage 1; M2: macrophage 2; MHC: major histocompatibility complex; PD1: programmed cell death protein 1; PDL1: programmed cell death ligand protein 1; TME: tumor microenvironment; Treg: T regulatory lymphocyte.

Over the past five years, immunotherapy has been proposed as a new therapeutic opportunity to fight cancers. The development of monoclonal antibodies against PD1 (pembrolizumab and nivolumab), PDL1 (atezolizumab, durvalumab and avelumab) and CTLA4 (ipilimumab) has created a new paradigm in the treatment of cancers by directly targeting immune cells, to stimulate an antitumor response, and not only cancer cells. Indeed, PD1 and PDL1 immune checkpoint blockades prevent inhibitory binding between surface proteins of the tumor and T cells. The use of anti-CTLA4 promotes the initiation and expansion of T cells by blocking the inhibitory interaction between the CD80 protein of antigen-presenting cells (APCs) and the CTLA4 protein of T cells. In addition, this antibody decreases the activation of Treg cells. Indeed, Treg cells exert an immunosuppressive function on effector T cells by constitutively expressing CTLA4 [139] (Figure 5A,B). However, while these treatments are efficient and very promising for some cancers (melanoma, kidney cancer, lung cancer, etc.), a large number of patients relapse or do not respond (Figure 5C). A study published in 2019 reported that after treatment with nivolumab alone, only 30% of patients with melanoma or renal cell carcinoma achieve a partial or complete response [18]. Innate (primary) or acquired (secondary) immune resistance and various side effects in patients are observed not only due to loss of neoantigen, defects in T cell function and lack of PDL1 expression in tumors but also due to genetic, epigenetic or transcriptomic alterations. Understanding them represents a major challenge for immunotherapy cancer research [140]. However, as shown in the previous section, there is a strong link between the metabolic profile of the TME and immune responses. Thus, a strategy to improve responses to immunotherapies could involve their combination with antimetabolic drugs to reprogram the immune status of the TME in favor of an antitumor response (Figure 6 and Table 1).

### 4.1. PI3K Inhibitors

PI3K is a crucial signaling pathway involved in cellular processes essential for cell survival, proliferation and differentiation. The tumor glycolytic switch related to the activation of this pathway is negatively associated in mice and humans with the antitumor response and the response to immunotherapy. Therefore, the PI3K–Akt–mTOR pathway could represent an attractive target for reversing the negative effects of metabolism and immunity in cancer. Nevertheless, the use of PI3K inhibitors as monotherapy has shown disappointing results. However, based on new therapeutic strategies, the combination of PI3K inhibition and PD1–PDL1 axis blockade has shown beneficial results. The loss of PTEN, a PI3K-inhibiting tumor suppressor often mutated in cancer, protects tumor cells from destruction by immune cells. Treatment of mice bearing *Pten*-null melanoma tumors in vivo with the PI3Kβ inhibitor GSK2636771 reduced Akt phosphorylation and activation of mTOR targets. In addition, this inhibitor combined with an anti-PD1 antibody markedly improved the survival and the levels of lymphocytes infiltrating the tumor (CD8^+^ T cells), as early as 15 days after treatment, resulting in a significant reduction in tumor mass [141]. However, this study did not show significant effect of the pan-PI3K inhibitor (BKM120) as described in more recent studies. This molecule has been tested as monotherapy in clinical trials for several years in different types of cancers and has shown promising results when combined with immunotherapy in in vitro or in vivo models. In bladder cancer and melanoma, studies have shown increased lymphocyte infiltration and cytotoxic functions of CTLs through the synergistic action of anti-PD1 antibody with BMK120 to reduce tumor growth [142,143]. Nowadays, many new anti-PI3K drugs are in development or being tested, offering promising strategies to increase patient survival. However, despite several studies, it has not yet been determined whether the antitumor mechanism induced by the combined anti-PI3K treatment with ICB is mainly due to an immunomodulatory metabolic effect or to an enhanced major proapoptotic effect.

### 4.2. Lactate Metabolism Modulation

Lactate is a well-known toxic byproduct produced by cancer cells to acidify the TME. Its production is inversely correlated with immune checkpoint inhibitor responses, and its targeting may therefore represent an effective therapeutic strategy for modifying the antitumor immune response. Thus, recent studies have shown that avoiding lactate accumulation by targeting MCTs or LDHA improves the efficacy of immunotherapy. Indeed, the survival of mice bearing LDHA-knockdown tumors was enhanced due to improved activation and expansion of CD8^+^ lymphocytes by secretion of IFN-γ [98]. In addition, Zappasodi et al. observed that blocking the transformation of pyruvate into lactate in combination with an anti-CTLA4 antibody decreased glucose uptake of tumor cells, whereas Treg cells increased glucose consumption, altering their suppressive capacity [100]. Lactate has been shown to serve as an alternative metabolite for Treg metabolism in the TME via its uptake by MCT1. MCT1-deficient Treg cells (Slc16a1^f/f^ FOXP3cre) exhibited less protumor functions due to an avid consumption of glucose to compensate for the oxidative metabolism related to the incorporation of lactate. Moreover, they exhibited an increase in the expression of PD1 suggesting the establishment of an environment conducive to immunotherapy. Indeed, the effects of the suppression of MCT1 in Tregs synergize with an anti-PD1 treatment to induce a complete regression in 37% of the mice bearing B16 cells [144]. Interestingly, elevated serum LDHA levels in patients with metastatic melanoma were correlated with a poor ICB response [145]. All these results provide evidence for therapeutic interest of lactate metabolism. Thus, AZD3975 is currently in phase I clinical trials to block the lactate transporter MCT1 in patients with solid tumors, diffuse large B cell lymphoma or Burkitt’s lymphoma (NCT01791595). Alternatively, neutralization of LDHA activity with GSK2837808A and 1-(phenylseleno)-4-(trifluoromethyl) benzene has shown promising effects on human cancer lines or preclinical models [146,147]. However, their implementation in clinical trials has not demonstrated a satisfactory effect due to limited membrane permeability and toxic effects [148].

### 4.3. AMPK Activation

AMPK can also be considered a key sensor to restore energy homeostasis in a tumor context. Identified for several years as a tumor suppressor, the activation of AMPK by metabolic stress leads to antiproliferative effects. Metformin, an antidiabetic drug that pharmacologically activates AMPK, potentiates anti-Warburg effects on various types of cancers (lymphoma, colon and breast cancers). Indeed, metformin alters the energetic activity of cancer cells by downregulating the expression of HIF-1α and mTOR, by limiting the activity of the protumor isoform HK2 and by inhibiting complex 1 of the electron transport chain [149,150,151]. Thus, metformin significantly reduces various energy-consuming cellular processes. In addition, the antitumor effect of AMPK is not limited to cancer cells but is also associated with systemic effects on immunity. At the myeloid level, metformin reduces the accumulation of MDSCs and induces the expression of cytokines promoting an M1 phenotype [152,153]. Chronic exposure to metformin in mice bearing head and neck squamous cell carcinoma (mEER) cells increases CD8^+^/Treg ratio and intratumor CD8^+^ infiltration to reduce tumor development [154]. Moreover, metformin not only potentiates the functional activity of CD8^+^ T cells but also increases their differentiation into memory T cells by preventing apoptosis [155]. Interestingly, in a murine model of 4T1 mammary tumor, activation of AMPK by metformin caused phosphorylation of Ser195 of PDL1 which induced abnormal glycosylation of the protein and its degradation by the proteasome, thereby increasing the cytotoxicity of CD8^+^ T cells [156]. These results, as well as those of a first small retrospective clinical study showing an overall improvement in survival, thus demonstrate the clinical relevance of combining metformin with ICB [157]. Pembrolizumab is thus combined with metformin in a phase I clinical trial in advanced melanoma (NCT03311308) and in phase II in head and neck squamous carcinoma (NCT04414540). In parallel, the combination of nivolumab and metformin is currently being tested in non-small-cell lung carcinoma (NCT03048500). However, as seen previously, AMPK, which inhibits mTOR and HIF-1α, also promotes the polarization of T cells into Treg lymphocytes and limits the production of IFN-γ, an anti-inflammatory cytokine secreted by Th1 lymphocytes [44,51]. Therefore, AMPK activators should not be used routinely for all cancers, and the TME should be analyzed beforehand to ensure that treatments will be of benefit.

### 4.4. Tryptophan–Kynurenine Pathway Blockade

The manipulation of the cellular metabolism of tryptophan could also be of therapeutic interest. IDO and TDO are the two limiting enzymes that degrade tryptophan to kynurenine. IDO is now known to be increased in many malignancies controlling immune tolerance. Although IDO has been associated with resistance to immunotherapy, the concomitant use of an IDO inhibitor with pembrolizumab (anti-PD1 antibody) in patients with unresectable melanoma has not provided benefit due to the establishment of immunosuppressive compensation by induction of TDO expression [158,159]. Therefore, even if new IDO1 inhibitors are under development, another innovative strategy is to degrade kynurenine into inert compounds in order to avoid the immunosuppressive kynurenine–AhR interaction. Thus, administration of a pharmacologically optimized enzyme (PEGylated kynureninase) to melanoma-bearing mice decreased plasma kynurenine levels without depleting TME tryptophan. In addition, this enzyme associated with immunotherapy improved the survival of mice by almost 50% [160].

### 4.5. Glutamine Pathway Inhibitors

Tumor remission and inflammation reduction may also be mediated by targeting glutamine metabolism. Glutamine, in high concentration in the tumor, is imported into tumor cells and converted into glutamate by glutaminase (GLS). Glutamate is then metabolized to support ATP production, redox balance and nucleotide synthesis and thus promotes tumorigenesis [161]. Thus, blocking glutamine metabolism may prevent the development of an immunosuppressive TME due to rapid malignant invasion. Several trials have been conducted or are underway on glutamine metabolic interventions associated with ICB. BPTES and CB-839, two allosteric inhibitors of GLS, limit cell growth in vitro and in vivo in many cancers. However, BPTES is less specific, less stable and less soluble than its homolog [162]. Moreover, CB-839 is being tested in ongoing phase I/II clinical trials in combination with nivolumab for the treatment of melanoma, clear cell renal cell carcinoma (CCRCC) and non-small-cell lung carcinoma (NSCLC) (NT02771626). The glutamine antagonist molecule DON, developed in the 1950s to limit the viability of tumor cells, has never been approved, due to significant side effects inducing neurotoxicity and gastrointestinal toxicity [163,164]. However, DON has shown significant effects on improving immunotherapy. The laboratory of J. Powell has designed a prodrug of DON (JHU083) which circulates intact and inert and is activated by enzyme cleavage only in the TME, thus limiting gastrointestinal toxicity. JHU083 has been tested in mouse models of colon cancer, lymphoma and melanoma. It not only inhibited tumor growth and altered the TME but also stimulated the generation of antitumor T cells by adapting their metabolism. Thus, the survival of the mice reached almost 100% when the molecule was associated with anti-PD1 or anti-PDL1 antibody treatment [165,166]. Finally, Byun et al. demonstrated that targeting glutamine decreased glutathione synthesis and upregulated PDL1 expression by altering SERCA activity, which activated the calcium/NFκB signaling cascade [166]. They showed that targeting glutamine metabolism synergizes the effects of ICB in mice with colon carcinoma.

### 4.6. Hypoxia and Adenosine Signaling Blockade

As mentioned previously, stabilization of HIF-1α contributes to increase adenosine levels deleterious to the activity of antitumor immune cells. Therefore, targeting immunosuppressive hypoxia–adenosinergic signaling may represent a novel therapeutic strategy associated with cancer immunotherapy. Indeed, the use of respiratory hyperoxia, to compensate hypoxia-mediated effects, decreased the concentration of extracellular adenosine which weakened Tregs and stimulated effector lymphocytes. In preclinical murine models of lung tumor immunotherapy, hyperoxia acted synergistically to promote tumor regression [167,168]. However, the results of this study were obtained with 60% oxygen supplementation for 72 h, which may raise questions about compliance with this therapy in patients already under heavy constraints. However, similar results were obtained with the use of A2AR antagonists. Targeting of the adenosine receptor restored T cell function by decreasing intracellular cAMP levels and improving tumor antigen cross-presentation by dendritic cells. Thus, increased infiltration of CD8^+^ T cells in the tumor was observed, which resulted in reduced tumor growth and metastatic dissemination [169,170]. The therapeutic potential of different A2AR antagonists tested as monotherapy or in combination with ICBs in clinical trials has shown promising results. Thus, ciforadenant (CPI-444) is being tested for multiple cancers (NCT02655822), AZD4635 has entered phase II in patients with prostate cancer (NCT04089553) and AB928 testing in pancreatic adenocarcinoma will be complete by 2022 (NCT03193190).

Another strategy is to target the two extracellular enzymes CD73 and CD39 which convert ATP into adenosine and facilitate immune escape by the tumor. It was first demonstrated by Stagg et al. in 2011 that CD73-deficient mice were resistant to tumorigenesis thanks to a decrease in FOXP3^+^ CD4^+^ cells [171]. Moreover, targeted therapy using the anti-CD73 monoclonal antibody MEDI9447 reduced immunosuppression in colon carcinoma. Furthermore, anti-CD73 therapy increased the therapeutic potential of anti-PD1 antibodies [172]. Likewise, targeting CD39 with the inhibitor POM-1 in a preclinical myeloma model decreased adenosine levels in the TME. The use of a combined treatment of POM-1 with an anti-CD73 antibody improved the antitumor efficacy of myeloid and lymphoid cells [173]. Therefore, CD39 and CD73 appear as potential therapeutic targets and are being tested in phase I clinical trials in combination with pembrolizumab on a wide range of cancers (NCT03454451). Since adenosine is produced from extracellular ATP, counteracting the elevation of extracellular ATP concentration in the TME would allow initial control of adenosine formation and thus maintain effective antitumor immunity. To achieve this, Mimoto’s team generated an antibody that binds to the human IL6 receptor only when ATP concentration is increased [174]. IL6R is an attractive target protein because many cancers (colon, ovary, liver, prostate, etc.) express the IL6 receptor and the release of IL6 cytokine stimulates tumor growth. The engineered ATP switch antibody does not bind to ATP present in low concentrations in normal tissue under physiological conditions. Mimoto’s team demonstrated that the tumor-specific distribution of the switch antibody drastically inhibited hepatic tumor volume in hIL6R transgenic mice bearing hIL6R-Hepa 1–6 tumors.

### 4.7. Dietary Modifications and Microbiota Modulation

Tumor cells, like other tissues in the body, consume plasma nutrients from the diet. However, because tumor cells have specific energy requirements, the plasma composition of the TME differs from that of healthy tissue [175]. Using dietary strategies in combination with chemotherapy/immunotherapy to control the availability of nutrients can increase the effectiveness of treatments and reduce their side effects. In preclinical studies, several types of diets that limit metabolites associated with malignancy have shown beneficial results in preventing cancer development. Caloric restriction (CR) is a chronic energy restriction that aims to decrease the availability of glucose due to its protumorigenic role. Thus, CR reducing caloric intake by 50% allows a 15% decrease in blood glucose levels in humans [176]. In mouse xenograft models (breast, prostate, pancreatic and liver cancers), studies have shown that CR prevents tumor development and metastasis dissemination by lowering the metabolic rate [177]. However, despite these anticancer effects, CR is not recommended in cancer patients with reduced physical condition due to a high risk of cachexia and sarcopenia. A better and growing strategy is to recommend a low-glucose but isocaloric diet such as the ketogenic diet (KD) characterized by high fat and low protein and carbohydrate contents. Since ketone bodies were not metabolized by tumor cells, KD inhibited tumor growth and improved survival in a malignant GL261 mouse model of glioma, in gastric adenocarcinoma and in LCaP prostate cancer mice [178,179,180]. Mechanistically, the decrease in circulating glucose decreased the systemic levels of insulin-like growth factor 1 (IGF-1) and thus the activation of the proproliferative PI3K signaling pathway [181,182]. In the context of immunotherapy treatment, KD enhanced innate and adaptive immunity in a mouse model of glioma by decreasing the expression of PD1, PDL1 and CTLA4 immune checkpoints and improving the infiltration and cytotoxic activity of TILs [183]. In addition, this diet also targets tryptophan metabolism by reducing plasma levels of kynurenine and thus attenuates the immunosuppressive effects of AhR activation [184]. Likewise, other dietary approaches such as diets low in protein or amino acids may also improve the responsiveness of the immune response. Indeed, dietary restriction in proteins and methionine reversed the immunosuppressive function of M2 macrophage towards an iNOS phenotype of M1 macrophage and a tumoricidal function with an increase in antitumor cytokines (IL-1β–IFN-γ–TNF-α). Moreover, it significantly improved the efficacy of immunotherapy [185,186].

Changes in our diet not only influence the TME but also affect the composition of the gut microbiota. Recent studies have highlighted the modulatory role of the gut microbiota on the antitumor immune response by targeting the PD1, PDL1 and CTLA4 immune checkpoints [187,188,189,190]. Gopalakrishnan et al. have shown that melanoma patients respond better to immunotherapy when their gut microbiome displays high diversity and abundance of Ruminococcaceae/Faecalibacterium [190]. Moreover, fecal transplantation of human responders in germ-free mice showed a better antitumor response and a decrease in tumor volume following anti-PD1 treatment compared to fecal transplantation of nonresponders. However, performing fecal microbiota transplantation (FMT) is complex, whether in terms of logistics, method of administration or the choice of healthy donors. Today, a single therapeutic indication for FMT is recognized for Clostridioides difficile infection, but various phase I clinical studies are underway in combination with immunotherapy in colorectal cancer (NCT04729322), advanced lung cancer (NCT04924374) and renal cancer (NCT04163289).

## 5. Conclusions

Immune cells undergo a metabolic switch to acquire their effector function against cancer. However, the metabolic deregulation and competition created by cancer cells in the TME disrupt their differentiation and alter many parameters such as their fitness, polarization, recruitment and survival. In the last few years, understanding the immuno-metabolic modifications in the TME has allowed the development of new therapies targeting the metabolism of tumor cells or immune cells favoring antitumor immunity. If today only a few patients respond effectively to immunotherapy, many clinical trials tend to show a synergistic effect of these ICB therapies in combination with metabolic drugs.

Other therapeutic strategies associated with the immune response are not developed in this review, such as cytokine therapy which is already commonly used to limit tumor growth by stimulating the cytotoxic activity of immune cells. However, today, due to severe inflammatory syndromes and limited efficacy in certain cancers (advanced kidney cancer, leukemia, myeloma and melanoma), only IL2 and IFN-α are administered to patients. Nevertheless, the clinical outcomes of IFN-γ, IL7, IL12, IL21 and IL15 are being evaluated in trials [191]. Adoptive T cell transfer is also a promising treatment for cancer. T cells from cancer patients are genetically modified to incorporate an artificial receptor, CAR, able to attack tumor cells. New CAR-T cell strategies are currently being developed to optimize CAR-T efficiency in a specific metabolic tumor context, but the first results in solid tumors are quite disappointing [192]. Finally, over the past decade, therapeutic cancer vaccines have gained renewed interest. This approach stimulates the immune response by delivering an exogenous tumor antigen specifically adapted to the patient’s cancer. Injected into the patient, they are captured by dendritic cells which become activated upon contact and are then able to exponentially activate T cells. While a vaccine (sipuleucel-T) is currently approved by the FDA for the treatment of metastatic prostate cancer, an immeasurable number of clinical trials are underway. Combinatorial strategies are also being optimized in mice to limit the failure of vaccine treatment. The combination of an antitumor vaccine with chemotherapy or with ICB or with CAR-T cells or with a metabolic immunosuppressive drug has shown promising results in promoting an antitumor microenvironment [193].

In conclusion, advances in preclinical and clinical studies have shown that metabolic interventions can improve the effectiveness of immune cancer therapies. Thus, continuing to better understand the metabolic needs of immune cells in the tumor microenvironment will be beneficial for the establishment of new cancer immunotherapy practices.

## Figures and Tables

**Table 1:** Nonexhaustive suggestions of potential metabolic targets that improve the antitumor immune response when associated with immune checkpoint blockades. AMPK: AMP-activated protein kinase; cAMP: cyclic adenosine monophosphate; CTL: cytotoxic T lymphocyte; PI3K: phosphoinositide 3-kinase; TME: tumor microenvironment; Treg: T regulatory lymphocyte.

Target	Metabolic Drug/Strategy	Cellular Effect	Immune Outcome
PI3K	GSK2636771 BMK120	Reduction of cancer cell glycolysis	Increase of lymphocyte infiltration and CTL cytotoxicity
Lactate	AZD3975 GSK2837808A 1-4-benzene	Decrease of lactate in the TME	Alteration of Treg activity and restoration of effector T cell functions
AMPK	metformin	Decrease of anabolic pathway activity	Increase of CD8^+^ infiltration and memory T cell differentiation
Tryptophan	PEGylated kynureninase	Degradation of kynurenine	Inhibition of Treg expansion
Glutamine	JHU083 CB-839	Reduction of cancer cell glutaminolysis	Increase of effector T cell proliferation
Hypoxia	hyperoxia	Decrease of intratumor hypoxia	Increase of lymphocyte infiltration
Adenosine	ciforadenant AZD4635 AB928 MEDI9447 POM-1 ATP switch antibody	Reduction of cAMP levels in the TME	Enhanced cytotoxicity of antitumor cells
